# Effect of Anatomically Realistic Full-Head Model on Activation of Cortical Neurons in Subdural Cortical Stimulation—A Computational Study

**DOI:** 10.1038/srep27353

**Published:** 2016-06-07

**Authors:** Hyeon Seo, Donghyeon Kim, Sung Chan Jun

**Affiliations:** 1 Gwangju Institute of Science & Technology, School of Electrical Engineering and Computer Science, Gwangju 61005, South Korea

## Abstract

Electrical brain stimulation (EBS) is an emerging therapy for the treatment of neurological disorders, and computational modeling studies of EBS have been used to determine the optimal parameters for highly cost-effective electrotherapy. Recent notable growth in computing capability has enabled researchers to consider an anatomically realistic head model that represents the full head and complex geometry of the brain rather than the previous simplified partial head model (extruded slab) that represents only the precentral gyrus. In this work, subdural cortical stimulation (SuCS) was found to offer a better understanding of the differential activation of cortical neurons in the anatomically realistic full-head model than in the simplified partial-head models. We observed that layer 3 pyramidal neurons had comparable stimulation thresholds in both head models, while layer 5 pyramidal neurons showed a notable discrepancy between the models; in particular, layer 5 pyramidal neurons demonstrated asymmetry in the thresholds and action potential initiation sites in the anatomically realistic full-head model. Overall, the anatomically realistic full-head model may offer a better understanding of layer 5 pyramidal neuronal responses. Accordingly, the effects of using the realistic full-head model in SuCS are compelling in computational modeling studies, even though this modeling requires substantially more effort.

Electrical brain stimulation (EBS) is an emerging electrotherapy spanning the whole field of functional neurosurgery: chronic pain^1^,^2^,^3^, rehabilitation^4^,^5^,^6^, Parkinson’s disease^1^,^7^,^8^, essential tremor^9^, and other brain disorders. EBS can be categorized commonly into invasive and non-invasive stimulation. Compared to non-invasive stimulation, invasive procedures enable one to selectively target specific regions of the cortex more effectively, and provide superior effects in such disabilities as chronic pain and movement disorders^10^,^11^,^12^. Invasive stimulation involves epidural cortical stimulation (ECS) through electrodes located above the dura mater, while subdural cortical stimulation (SuCS) is accomplished by placing electrodes below the dura mater. SuCS, in particular, has some advantages, including the fact that it is easy to target the cortical surface with less intense current than in ECS and that it can be used as an alternative approach when ECS fails in patients with advanced cortical atrophy due to duro-cortical separation. Furthermore, SuCS has additional benefits compared to deep brain stimulation (DBS) in some conditions, such as central pain; it also appears to be more cost-effective^11^.

There are many stimulation parameters in SuCS, such as electrode shape and placement, stimulus amplitude, frequency, polarity, pulse width, etc., that must be optimized in a reasonable manner. Thus, a large number of possible combinations of these stimulation parameters may induce different therapeutic outcomes, thereby making it more difficult to determine the optimal parameters. A computational study is one of the most useful approaches in investigating the influence of these various parameters. Such investigations have primarily used two kinds of volume conduction models; these are represented by a simplified partial-head model^13^,^14^,^15^,^16^,^17^,^18^,^19^ or a full-head model^20^,^21^,^22^,^23^,^24^,^25^,^26^ that considers the complex geometry of the brain. The simplified partial head model is an extruded slab model that represents a part of the brain, typically the precentral gyrus area. This model is highly efficient in terms of computational time and allows conjectures of stimulus-induced neuronal responses, because it could be coupled with compartmental neuronal models in a more straightforward manner due to the simplicity of its geometry^13^,^14^,^16^,^19^,^27^. Therefore, it may be possible to perform direct observations of the activation of cortical neuronal models with various extracellular information. However, using the extruded slab model could induce inaccurate predictions of neuronal responses because of possible modeling errors. The full-head model is anatomically realistic and represents the entire geometry of the head while also providing precise information about head anatomy obtained from magnetic resonance (MR) imaging. The anatomically realistic head model is suitable for detailing various anisotropic information and estimating stimulus-induced electric field (EF) or current density (CD). Due to the fact that the detailed geometry of the head increases realism, the anatomically realistic head model may have some benefits in improving the estimation of stimulation targets and optimizing stimulation parameters^24^,^28^,^29^. Therefore, recent studies have focused on estimating stimulus-induced EF/CD distribution using the anatomically realistic head model^20^,^21^,^22^,^23^,^24^,^25^. However, they have inferred neuronal excitability based on EF/CD, which is a less efficient estimation of stimulation effects, as cellular effects vary depending on neuronal morphology and stimulation parameters, such as intensity and direction^16^,^27^,^30^,^31^,^32^.

In response to such needs, our group investigated the effects of SuCS on cortical neurons using the anatomically realistic head model^26^, and then investigated the effects of anisotropic conductivity. However, incorporating compartmental neuronal models into the complex head model was challenging, as it incurred huge computational costs. Even building the model may take a significant amount of manual work and require various imaging resources^24^,^29^. Hence, it would be useful to know how significantly the anatomically realistic head model improves the estimation of cellular mechanisms, despite the complex and large computation processes required. Several studies have examined the effects of different volume conductor models on the EF/CD induced during transcranial magnetic stimulation^29^, deep brain stimulation^33^, and SuCS^34^. They reported that there were substantial differences between the simplified partial head model and the realistic full head model. However, these effects were inferred from stimulus-induced EF/CD as the stimulus-induced neuronal responses have not yet been investigated.

In this regard, by comparison to the simplified partial-head model, the effects on activation of cortical neurons were investigated in the anatomically realistic head model by incorporating layer 5 pyramidal neuronal models, and our group presented the results at EMBC 2013^35^. In our preliminary work, we modeled a small number of neurons, adjusted their locations manually, and then investigated the neuronal activation over the stimulus polarities in these head models. This study was limited because it did not consider anisotropic conductivity properly in the anatomically realistic head model, and it was simulated with too few neurons to represent the precentral gyrus. For these reasons, in this work, we constructed an anatomically realistic head model with anisotropic conductivity acquired from diffusion tensor (DT) imaging and a large number of neurons that represented two kinds of pyramidal neuronal models (layers 3 and 5). Those pyramidal neurons were then distributed uniformly within the two head models in order to compare their neuronal activation. Activation of neuronal models was observed with the three stimulus polarities of anodal, cathodal, and bipolar stimulation. In addition, excitation thresholds that represent the stimulus amplitude needed to trigger a neuron’s action potential and the site of that action potential initiation were analyzed. Thus, our focus on the discrepancies between the two common head models was designed primarily to determine whether or not the anatomically realistic head model yielded better estimation of cortical neuronal activation, which is not yet understood clearly. For this reason, this work may be considered an extended version of our conference article^35^.

## Results

To investigate the influence of head model geometry on neuronal activation, we applied a 100 μs monophasic rectangular pulse to the motor cortex by varying stimulus polarity (anode and cathode). The excitation thresholds necessary to evoke action potential of neuronal models were then measured in both the extruded slab and anatomically realistic head models over various stimulus amplitudes. Due to the advantages that this process held for our simulation study, we considered stimulus amplitudes up to 100 mA, which is far higher than the typical acceptable range of under 20 mA^4^,^5^,^6^,^10^,^36^. In this way, it is possible to observe the trends in neuronal activations.

**Figure 1:**
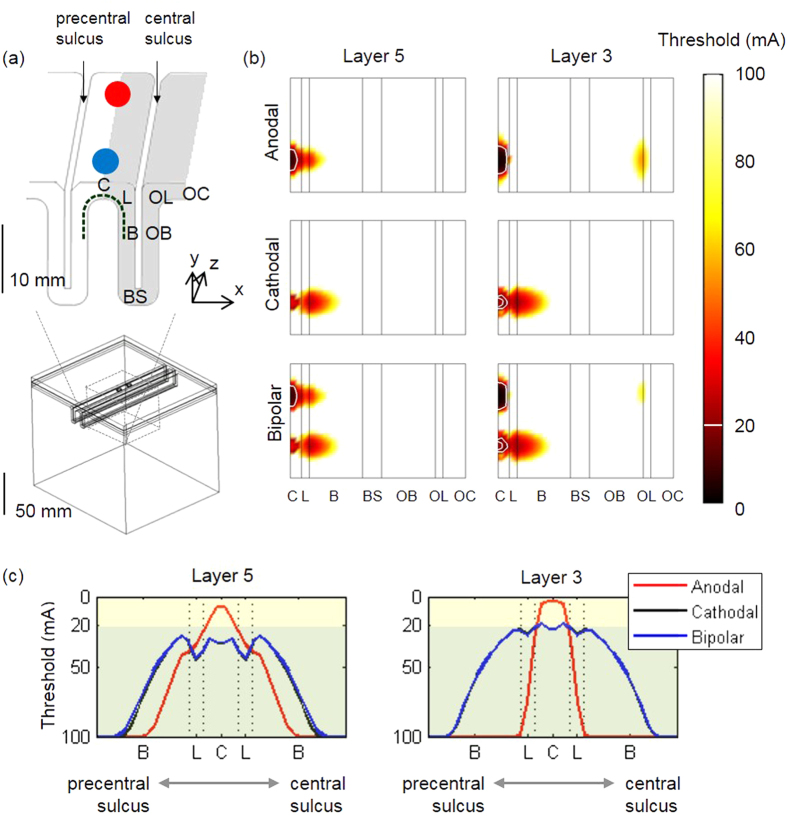
The spatial extent of the excitation thresholds in the extruded-slab model. (**a**) Because of symmetric geometry, neuronal models were distributed within half of the cortex, which is represented as a gray colored region; in the x-y plane, the gray matter (GM) is classified according to the cortex location and orientation. The crown (C) is the region directly under the electrode, and the lip (L) and bank (B) are located along the fold on gyrus; the bottom sulcus (BS) lies beneath the central sulcus; the postcentral gyrus consists of the opposite bank (OB), opposite lip (OL), and opposite crown (OC). (**b**) The spatial extent of the thresholds stretching the surface from the gray colored region in (**a**) in the x-direction is shown. The horizontal axis represents the abbreviation of the different region of the cortex; the white contour lines represent excitation thresholds <20 mA. (**c**) Excitation thresholds along the curve (depicted as a dotted curve in (**a**)) from the bank of the precentral sulcus to the bank of the central sulcus under the active electrode (blue circle).

**Figure 2:**
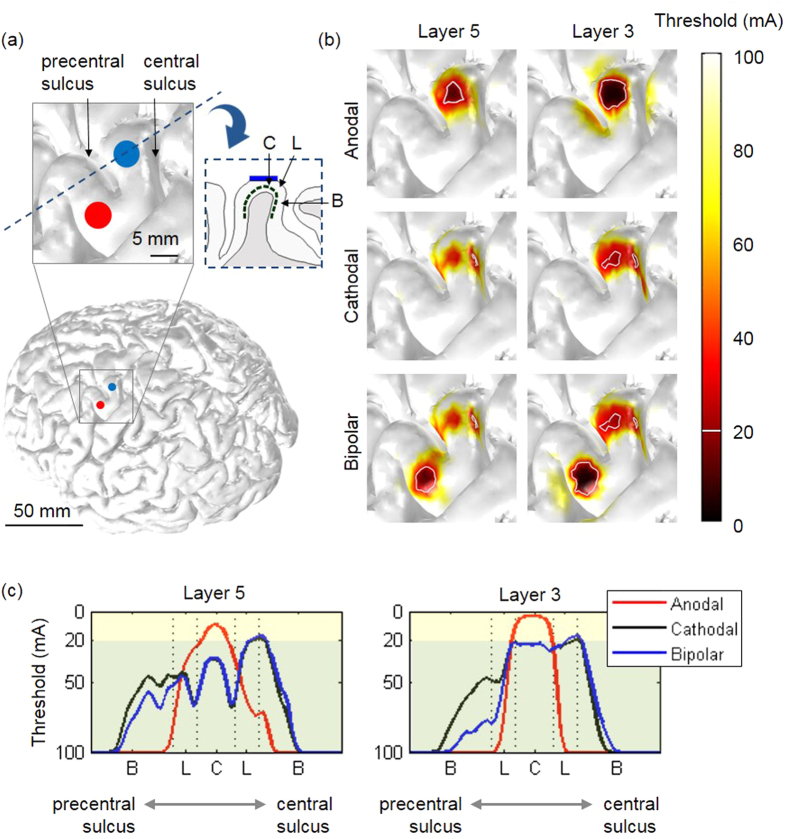
The spatial extent of the excitation thresholds projected on the cortical surface in the anatomically realistic head model. (**a**) Electrodes were placed on the precentral gyrus representing the hand area; the left inset represents the expansion of the GM surface and right inset indicates the cross-section perpendicular to the top electrode (following blue colored dotted line) with the cortex location of the crown (C), lip (L), and bank (B). In bipolar stimulation, the upper electrode represents the cathode (blue), while the bottom electrode is the anode (red). For convenience, monopolar stimulation was applied using only the upper electrode; (**b**) this is the spatial extent of the thresholds of L5 and L3 neurons in the anatomically realistic head model; the white contour lines represent excitation thresholds <20 mA. (**c**) Excitation thresholds along the curve (depicted as a dotted curve in the right inset of (**a**)) from the bank of the precentral sulcus to the bank of the central sulcus under the active electrode (blue circle).

Figures 1 and 2 illustrate the spatial extents of the excitation thresholds in the extruded slab and anatomically realistic head models, respectively. They show similar threshold patterns and spatial extents. On the whole, during anodal stimulation, neurons in the crown beneath the electrode have generally had the lowest thresholds, while the lip to upper part of the bank and the opposite lip were excitable in L5 and L3 neurons, respectively. Cathodal stimulation activated neurons in the deeper bank compared to anodal stimulation, while bipolar stimulation seemed to be a superposition of the two monopolar stimulations: cathodal and anodal. Differences between the two models were characterized in the cathodal stimulation. In the anatomically realistic head model, the patterns of spatial extent seemed asymmetric. In both L5/L3 neurons, the bank along the central sulcus was observed to be most excitable area, while the bank along the precentral sulcus was not (Fig. 2(c)). An activated area with stimulus amplitude lower than 20 mA (represented by white contour lines (b) and highlighted by light yellow shaded region (c) in Figs 1 and 2) was focused around even bank during cathodal stimulation in the anatomically realistic head model. However, in the extruded slab model, the crown was found to be the most excitable area regardless of stimulus polarities, and it had symmetric patterns of excitation threshold due to its inherently symmetric geometry.

**Figure 3:**
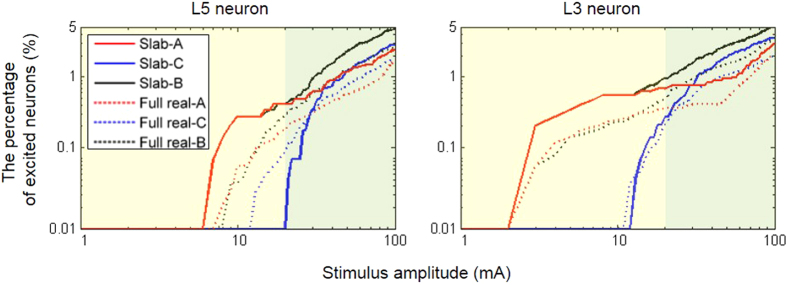
The relative ratio (%) of neurons excited with three different polarities: (anodal (A); cathodal (C), and bipolar (B) stimulations). Layer 5 (L5) and layer 3 (L3) pyramidal neurons are compared within the extruded slab model (slab) and anatomically realistic head model (full real); neurons that evoke action potentials within 20 mA are highlighted in light yellow.

For further investigation, the ratio of excited neurons to total neurons was observed for three different anodal/cathodal/bipolar stimulations (Fig. 3). When we focused on L5 neurons excited under the stimulus amplitude of 20 mA (represented by the light yellow shaded region in Fig. 3), the extruded slab model induced a much higher percentage of excited neurons than did the anatomically realistic head model during anodal stimulation and a smaller percentage during cathodal stimulation. It did not show substantial differences in L3 neurons during cathodal stimulation. Overall, it was evident that the extruded slab model yielded notably higher percentages of excited neurons than did the realistic head model during anodal stimulation, which suggests that the extruded slab model may overestimate the stimulation effects. We observed that anodal stimulation started to excite neurons at lower stimulus amplitudes than did cathodal stimulation; however, this was reversed at high stimulus amplitudes. This behavior was observed in both L3/L5 neurons and both head models (over about 25–30 mA in L3/L5 neurons in the anatomically realistic head model; and over about 30 mA in L3 neurons and about 50 mA in L5 neurons in the extruded slab model). Overall, bipolar stimulation excited more neurons than did the others because it seemed to be a summation of cathodal and anodal stimulations.

For the given stimulus polarity and cell types (L5 or L3), we summarized the minimum excitation threshold required to evoke neuronal activity and the area where excited neurons are located, which are tabulated in Table 1. Consistent with previous findings from the ratio of neurons excited over varying stimulus amplitudes (Fig. 3), we note in Table 1 that anodal stimulation had substantially lower thresholds than did cathodal stimulation. As shown in previous results (Figs 1 and 2), bipolar stimulation was shown to be a simple summation of anodal and cathodal stimulations. Thus in bipolar stimulation, the minimum thresholds under the active electrode were identical to those of anodal stimulation, and under the ground electrode they had the same minimum thresholds to cathodal stimulation. During anodal stimulation, the minimum thresholds were increased from the crown to the bank along the fold of gyrus (the minimum thresholds in the lip and bank were 17 and 23 mA for L5 neurons, and 7 and 55 mA for L3 neurons), while during cathodal stimulation, neurons in the bank had lowest threshold and the minimum thresholds in the crown and lip were comparable (the minimum thresholds in the crown and lip were 21 mA for L5 neurons, and 14 and 15 mA for L3 neurons). It is interesting that, during cathodal stimulation, the minimum threshold of L5 neurons in the anatomically realistic head model was substantially lower (60%) than in the extruded slab model. In the extruded slab model, neurons excited at the lowest threshold were located uniformly on the crown, regardless of polarities, while in the anatomically realistic head model, cathodal stimulation activated the bank at 11 mA (L3 neurons) or 13 mA (L5 neurons), which are the minimum excitation thresholds.

**Figure 4:**
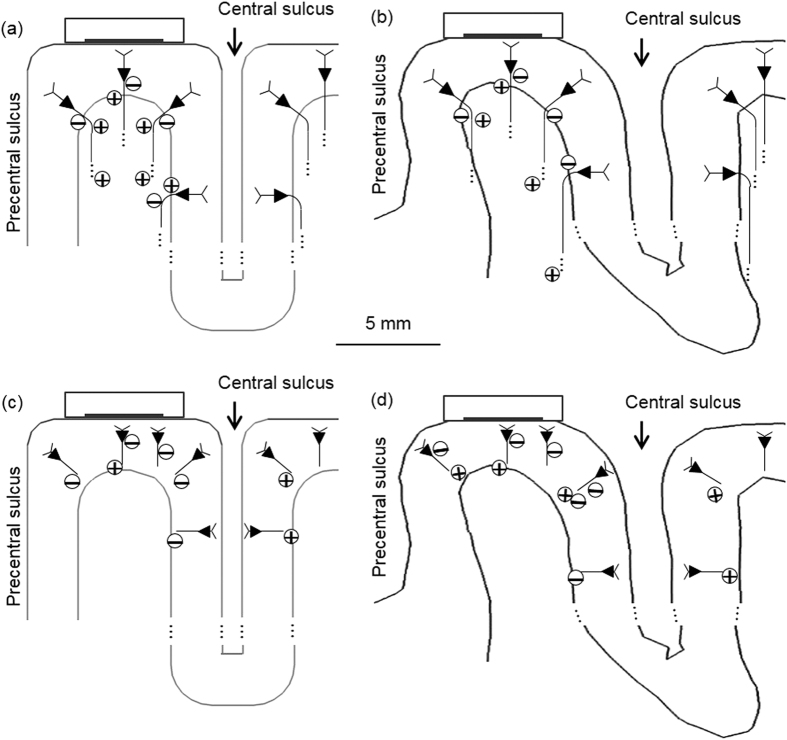
Initiation site of action potential over anodal (+) and cathodal (−) stimulation. (**a**) L5 neurons and (**c**) L3 neurons in the extruded slab model; (**b**) L5 neurons and (**d**) L3 neurons in the anatomically realistic head model.

The site of initiation of the action potential evoked by extracellular stimulation was examined by recording membrane potentials at several different locations on L5 and L3 neurons (Fig. 4). We observed the initiation sites of L5/L3 neurons that evoked action potentials under the 100 mA stimulus amplitude and found that they varied according to the neuronal model’s location, morphology, and stimulus polarities. The axon terminal was frequently excited in L3 neurons, which may be due to the finite length of L3 neurons within gray matter (GM). The axon at the boundary between GM and white matter (WM)—where conductivity changes abruptly—was often observed as the initiation site for L5 neurons. Cathodal stimulation evoked the excitation of L3 neurons on the crown (perpendicular to the electrode) at the initial segment, while anodal stimulation evoked L3 neurons at the axon terminal. However, L5 neurons on the crown were evoked at the boundary between GM and WM for both anodal and cathodal stimulations. In the lip and the bank following the fold of the gyrus, the initiation site for L5 neurons in the anatomically realistic head model was the axon terminal during anodal stimulation and the boundary between GM and WM during cathodal stimulation. In the extruded slab model, neurons in both the lip and the bank showed inconsistent initiation sties.

The major differences between the two head models were likely due to the asymmetric geometry of the anatomically realistic head model; differences were observed around the lip, in particular. During anodal stimulation, initiation sites of L5 neurons at the lip were located at the bends in the axons on the side of the precentral sulcus, as well as at the axon terminals in the direction of the central sulcus. These asymmetric initiation sites were also observed around the lip in L3 neurons.

## Discussion

Computational brain modeling could be a prerequisite in determining the optimal parameters necessary to design efficient electrotherapy treatments^11^,^28^. Thus far, many computational studies have been extrapolated cellular targets of electrical stimulation by stimulus-induced electric fields. For precise patterns of electric fields, the importance of the anatomically realistic head model has emerged for individualized modelling according to each subject by incorporating magnetic resonance imaging (MRI) and diffusion tensor MRI (DT-MRI); it has also been investigated actively by noninvasive electrical stimulation^20^,^21^,^23^,^24^,^28^,^37^,^38^. Edward *et al*.^37^ constructed three different head models using each subject MRI and then validated a computational model for estimating the stimulus-induced electric field in human transcranial stimulation. They revealed that the model data were consistent with the motor responses across subjects. Furthermore, operating under the assumption that induced electric field is comparable to cortical activation, they found that the model predicted a significant difference (more than twofold) in the induced electric field on the primary motor cortex across subjects. The computation study of Truong *et al*.^38^ reports the comparable variation across subjects and describes the importance of the construction of an individualized anatomically realistic head model; however, only a few studies of invasive electrical stimulation have been performed using the anatomically realistic head model^22^,^34^,^35^. It is understood that invasive stimulation may stimulate a relatively smaller focal area of the brain than noninvasive stimulation; thus, the use of a more complex head model representing the whole brain area may actually be relatively less demanding.

In general, the degree of activation can be approximated by employing the stimulus-induced electric field or current density. In particular, the electric field is known to relate directly to neuronal activation under the quasiuniform assumption^28^. Another way to estimate the extent of neuronal activation is to compute the second derivative of the potential along the direction of the neurons (directional derivative) which is known as the activating function^39^. However, these methods may be less accurate in estimating neuronal activation because neuronal morphologies and their electrical properties are not fully considered.

The activating function shows a simple reversal in signs between anodal and cathodal stimulations because the brain model is linear with respect to electric potential. Due to such limitations of simple extrapolations, a compartmental neuronal model is incorporated into computational brain models in order to yield more accurate estimations of neuronal activation^13^,^14^,^16^,^19^. However, in previous studies, the compartmental neuronal models were coupled with the extruded slab model, which is a very simplified and less accurate model that may lead to inaccurate estimations of neuronal activation due to a modeling error.

The insertion of electrodes into the anatomically realistic head model is difficult in terms of generating the computational mesh due to irregular brain tissue morphologies. In this work, the insertion of electrodes into the realistic head model was attempted and an anatomically realistic head model was eventually constructed. We then incorporated numerous numbers of compartmental models of L5/L3 pyramidal neurons combined with the anatomically realistic head model, enabling us to directly investigate the effects of head model mismatch. In this work, this model mismatch was analyzed in terms of four distinct aspects: 1) spatial extent of threshold, 2) the percentage of excited neurons, 3) the minimum threshold, and 4) initiation site of action potentials.

First, in the spatial extent of excitation thresholds, asymmetric patterns in the anatomically realistic head model were observed compared to the simplified extruded slab model. Specifically, during cathodal stimulation, L5 neurons in the bank close to the central sulcus were activated more than the L5 neurons in the precentral sulcus, although this is not illustrated clearly in Fig. 2. The bank along the central sulcus is known to be the anatomical location of the motor area of the hand^40^, which is the most critical target area of invasive stimulation^5^. As a result, it is believed that the anatomically realistic head model may estimate cellular targets more accurately than the extruded slab model.

Second, regarding the percentage of excited neurons, the extruded slab model induced more depolarization (a higher ratio of excited neurons) during anodal stimulation and more hyperpolarization (a lower ratio of excited neurons) during cathodal than did the anatomically realistic head model. This result was expected based on our earlier study^34^, where we reported the current distribution difference in SuCS between the realistic head model and the extruded slab model. The extruded slab model was more likely to overestimate neuronal activation in SuCS than was the anatomically realistic head model. However, this work may be the first report that, at the neuron level, the activation effects of cortical neurons in the extruded slab model may be relatively overestimated compared to the anatomically realistic head model.

Third, regarding the minimum threshold, the model discrepancy was notable during cathodal stimulation. The crown was the area where the top of gyrus was activated most easily in the extruded slab model, while the bank was activated well in the anatomically realistic head model. In addition, regarding the initiation site of action potentials, the two head models yielded similar behaviors on initiation sites, except for in the lip and in the bank. This discrepancy may come from asymmetry in the anatomically realistic head model.

We observed that differences in the head models yielded variations in excitation thresholds; the distinctions were more notable in L5 neurons than in L3 neurons. We understand that L5 neurons are affected significantly by the geometry of the brain model because of its longer axon that extends into the WM, while L3 neurons are located within the GM.

The simulated pyramidal neuronal responses showed an agreement with the empirical findings from previous research. Gorman^41^ reports that, at the threshold of stimulus amplitude necessary to elicit neuronal responses, a direct wave was elicited during anodal stimulation, while cathodal stimulation produced an indirect response. As stimulus amplitude increased up to supramaximal intensity, cathodal stimulation produced a greater response in the direct and indirect activations than anodal stimulation. In this work, we investigated the individual neuronal models which enabled us to observe the direct response. At the stimulus amplitude where anodal stimulation evoked action potentials of L5 and L3 neurons, cathodal stimulation did not excite any neurons (Fig. 3). Interestingly, there were points of intersection between anodal and cathodal stimulations that were about 25–30 mA in the anatomically realistic head model and about 30 or 50 mA in the extruded slab model; at a higher stimulus amplitude than those points, cathodal stimulation produced a higher percentage of excited neurons than did anodal stimulation.

The minimum excitation thresholds (Table 1) are in accordance with previous results that show anodal stimulation activated neurons at lower stimulus amplitudes than did cathodal stimulation^2^,^13^,^14^,^16^,^41^,^42^,^43^. Bipolar stimulation appeared to be a superposition of anodal and cathodal stimulations, as shown in Figs 1 and 2 and Table 1. This is consistent with the results of previous computational studies^2^,^13^,^14^,^16^,^19^, which report that bipolar stimulation with an inter-electrode distance greater than 10 mm produces little interference. In this work, inter-electrode distances are greater than 10 mm in both head models.

Initiation sites of action potential varied according to the position of the neuronal models, stimulation polarity, and relative direction of the stimulus-induced electric field^16^,^44^. According to our computational study, L3 neurons were initiated easily at the axon terminal, while the action potential of L5 neurons was initiated frequently at the boundary between the GM and WM, where conductivity changes abruptly^45^. Furthermore, we observed that during cathodal stimulation, initiation sites were pinpointed more in areas closer to soma than were those produced by anodal stimulation; these findings are relevant to existing studies^13^,^14^,^16^,^19^,^46^ which report the longer latency in cathodal stimulation compared to anodal stimulation in experimental studies and investigate the stimulation effects on invasive approaches in computational studies.

In this work, the neuronal model was based on properties from the cat visual cortex due to limited knowledge about properties and morphologies of most human cortical neurons. Despite this uncertainty in the neuronal models, it was observed that the computational results were quite reasonably consistent with experimental data. The compartmental models of pyramidal neurons used in this work were incorporated also on several computational studies to investigate stimulation effects. Wongsarnpigoon *et al*.^16^ studied the effects of electrode position and geometry on neuronal activation in the epidural cortical stimulation. Prior to the computational study, they validated compartmental pyramidal neuronal models located in the crown by comparing with these models with experimental data. Kamitani *et al*.^47^ constructed neocortical neuronal models in TMS and showed brief firing followed by a silent period of duration, which was comparable to experimental data of TMS. Thus, these studies provide a valid rationale of computational results that well match those in this study.

It is challenging to make direct comparisons between the extruded slab model and anatomically realistic head model; however, as both models are the most commonly used in invasive CS studies, we analyzed the responses of neuronal models induced by differences in the head models and then observed the discrepancy in those neuronal responses. Grant and Lowery^33^ proposed an ellipsoid-shaped realistic head model that sets the ground on the bottom, where the reference electrode can be located. This ellipsoid realistic head model was compared with a cubit model that set the ground at all exterior boundaries. They found that electrical grounding and the finite conducting volume of the head had considerable effects on the electrical potential, electrical field, and activating function. This may imply that the effects of different head models may be due to the grounding effect. In this regard, we investigated grounding effects under a more extended extruded slab model and set the reference electrode on the bottom. This model was proposed by Zwartjes *et al*.^19^ and considers boundary conditions based on a realistic head model^33^. Then, we compared two kinds of extruded slab models during monopolar stimulation and found that the two models had comparable excitation thresholds, with an average 2.33% difference.

Other critical model parameters that may have a significant impact on any discrepancy between the extruded slab and anatomically realistic head models include the model anisotropy of conductivity and geometry. First, we investigated whether or not anisotropic conductivity has a significant effect on cellular targets. Two computational head models were set to isotropic conductivity (WM conductivity was assigned to 0.126 S/m^21^), and then stimulation changes induced by tissue anisotropy to the equivalent isotropic model were assessed. The anisotropic models yielded substantially different results compared to the isotropic models. As tabulated in Table 1, during anodal stimulation, the minimum thresholds were 3 mA in the anatomically realistic head model and 7 mA in the extruded slab model; cathodal stimulation had a much higher value of the minimum threshold compared to anodal stimulation. In contrast to the results from anisotropic models, results of the model being set to the isotropic conductivity showed minimum thresholds were much higher during anodal stimulation than during cathodal stimulation. The minimum thresholds in the extruded slab model were 23 mA and 15 mA in anodal and cathodal stimulations, respectively. In the anatomically realistic head model, they were 19 mA and 7 mA, respectively. Interestingly, these results from isotropic models were irrelevant to the experimental ones that showed anodal stimulation activated neurons at lower stimulus amplitude then did cathodal stimulation^2^,^41^,^42^,^43^. Furthermore, isotropic models excited far more neurons during anodal stimulation and fewer neurons during cathodal stimulation compared to the anisotropic model^26^. As a result, we concluded that anisotropy of conductivity, as well as the complexity of head model geometry, are major factors that produce the discrepancies between the extruded slab and anatomically realistic head models.

The positions of the L5/L3 neurons may influence our computational results. In order to estimate these effects on neuronal activation, we slightly disposed the neuronal models; positions of L3/L5 neurons were shifted upward or downward by 1 mm in the perpendicular direction to the cortical surface. We observed that L5 neurons, which are activated under 100 mA, showed an average of 6.36% differences and L3 neurons had an average of 7.3% differences during monopolar stimulations. In addition, in order to briefly investigate the effect of neuron orientation, we rotated it up to 90 degrees in increments of 30 degrees, since the morphology of dendrites is not symmetric. Slight change was observed, with a maximum of 2.8% differences in both L5 and L3 neurons according to the rotation of the dendrites. These investigations showed that the positions of neurons had mild effects on neuronal activations; however, spatial extent of thresholds, minimum threshold, or patterns of percentage of excited neurons were not substantially varied.

Through this computational study, we revealed that the anatomically realistic head model may be recommended as an effective, beneficial model used to investigate cellular targets and detailed neuronal responses. This recommendation comes despite the fact that relatively smaller and more focalized areas may be involved in the investigations as well as construction of the realistic head model requires substantial efforts. The increased effort might be due to incorporating precise information of model anatomy and anisotropic conductivity acquired from MRI and DT-MRI. For further study, we plan to model communicating populations of neurons and generate indirect responses following synaptic excitations. It is expected that the advantages of the anatomically realistic head model will be more considerable in this further study because of the larger area that may be involved in neuronal excitation.

We know that a target-neural element is necessary to improve clinical results, and it is believed that targeted neural elements may vary according to electrode positions, geometry, and polarities^16^. For example, the analgesic effect of motor cortex stimulation is related to the specific neurons excited in the superficial cortical layer and not to the intensity of the stimulus^3^. In the same context, effects are also related to electrode locations: electrodes placed perpendicular to the precentral gyrus were recommended in order to improve therapeutic effects^2^,^3^, while other studies have demonstrated improved outcomes with electrodes oriented parallel to the precentral gyrus^48^. In our work, we demonstrated that the anatomically realistic head model is more useful in investigating the target area and detailed responses of neuronal activation. Further studies should focus on parametric analyses using the anatomically realistic head model rather than the extruded slab model.

## Methods

### Two head models of SuCS

**Figure 5:**
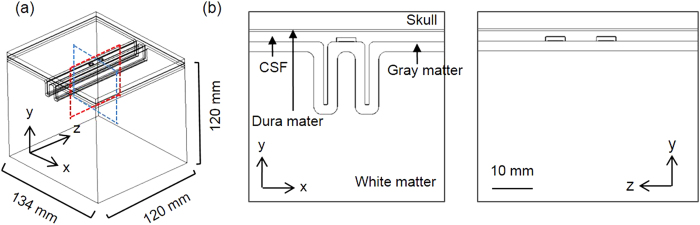
The shape of the extruded slab model. (**a**) Three-dimensional extruded slab model representing motor cortex, and (**b**) a cross-section of the model passing through electrodes.

Two volume conduction head models for SuCS (a simplified partial head and an anatomically realistic full head), including stimulus electrodes, were developed to investigate the effects of head model geometry on the activation of cortical neurons. First, we constructed the simplified partial head model (extruded slab), which has a uniform brain geometry along the z-axis and an intrinsic two-dimensional geometry, as illustrated in Fig. 5. Details were described previously (Seo *et al*.^35^). The dimensions of the precentral gyrus (8.5 mm) was reduced in the present model to more closely match the dimensions of the precentral gyrus in the anatomically realistic head model. This model is restricted to the precentral gyrus and its surrounding sulci and gyri. Two covered, disc-type electrodes, 13 mm apart, were placed on the cortex parallel to the precentral gyrus. These electrodes were designed by considering the clinical use of strip-type electrodes and the ease of modeling the electrodes in the anatomically realistic head model^22^. In a clinical situation, a pulse generator (reference electrode) would be implanted in the pectoral region; however, that was not a possible consideration in this model. It was assumed that all boundaries, except the upper boundary of the skull that was set as an electric insulator, are grounded during monopolar stimulation (anodal or cathodal).

**Figure 6:**
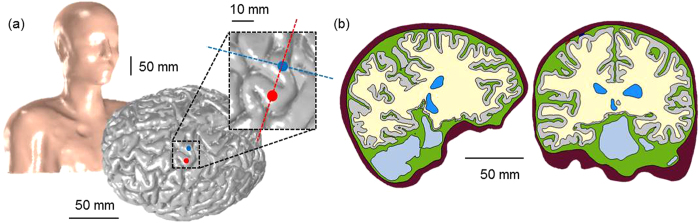
The shape of the anatomically realistic head model. (**a**) The complex, whole-head model (left) and placement of subdurally implanted electrodes on the gray matter (right), and (**b**) the cross-section perpendicular to the top electrode (blue circle; left) and parallel (right) to the two electrodes.

The second model is the anatomically realistic head model obtained from MRI data (Fig. 6). A whole upper body from the Visible Human Project of Korea^49^ and the brain MRI of one living human were obtained from the SimNIBS^24^. We note that this human MRI data is anonymous and publicly accessible. For this reason, this study did not require Institutional Review Board (IRB) approval from the Gwangju Institute of Science and Technology (GIST).

The anatomically realistic full head model was constructed using well-known tools: FreeSurfer^50^,^51^, FMRIB FSL^51^, and Seg3D^52^ (refer to Kim *et al*.^34^ for details). Then, two electrodes were designed with same configuration as those in the simplified partial head model and placed on the precentral gyrus representing the hand area (Fig. 6(a)). One reference electrode (height = 12 mm; diameter = 11.5 mm) was attached to the chest. Finally, optimized volumetric mesh was generated using iso2mesh^53^ and TetGen^54^.

These 3D computational head models were implemented in COMSOL Multiphysics (v4.3b, COMSOL, Inc., Burlington, MA, USA) and solved using the finite element method (FEM). The number of total tetrahedral elements in the simplified partial and anatomically realistic head models was approximately 1.2 million and 8.8 million, respectively. The bi-conjugate gradient method (a relative tolerance of 1 × 10^−6^) with preconditioning of an algebraic multigrid was applied.

### Conductivity assignment

Anisotropic conductivity was assigned to the WM only, and other segmented layers of the head models were set to isotropic conductivity (in S/m)^13^,^20^,^55^,^56^,^57^,^58^: scalp: 0.465; skull: 0.01; dura mater: 0.065; CSF: 1.65; gray matter: 0.276; electrode: 9.4 × 10^6^; and substrate: 0.1 × 10^−9^. For more details on the conductivity assignment, refer to Seo *et al*.^26^,^35^.

In the simplified partial head model, we assumed that the dominant direction of the fibers was perpendicular to the skull (y-axis). In contrast, in the anatomically realistic head model, the major direction of the fibers was stretched, reflecting the complex geometry of the brain. Therefore, anisotropic information was acquired from diffusion tensor magnetic resonance imaging (DT-MRI) under the assumption that conductivity and diffusion tensors share the same eigenvectors^59^.

In this work, we adopted the eigenvector from DT-MRI and set the same eigenvalues as those in the extruded slab model, such that the longitudinal direction was set to 1.1 S/m with the transverse direction at 0.13 S/m^15^,^60^. The tensor representation of WM anisotropy is given by


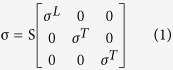


where S is an orthonormal eigenvector matrix and *σ*^*L*^ and *σ*^*T*^ are conductivities in the longitudinal and transverse directions.

### Compartment models of pyramidal neurons

We developed two types of layer 5 (L5) and layer 3 (L3) pyramidal neuronal models because pyramidal neurons are known to be the primary activators of the corticospinal tract and may provide the main input to the direct pathway^19^,^41^. Their morphology and electrical properties were taken from the cat visual cortex^61^ and then modified by lengthening them 60% in order to fit human brain geometry^16^.

Those neuronal models were indirectly coupled with the head models, so that electric potentials calculated in each head model were, as an extracellular stimulation, applied to neuronal models with a 100 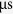 monophasic pulse. We then analyzed the threshold for direct activation and the sites of action potential initiation for the two different models by varying stimulus polarities and amplitudes. We note that the simulations for neuronal models were performed in the NEURON environment^62^.

**Figure 7:**
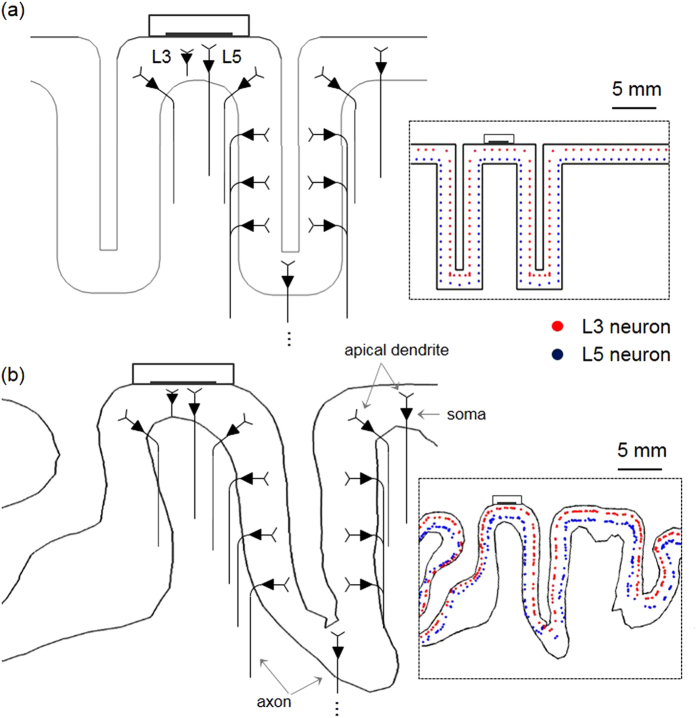
Schematic view of distribution of the compartmental pyramidal neuronal models. In the cross-sections perpendicular to the electrode, neurons represent relative orientations according to their locations. However, only a few neurons among the uniform neuronal models are shown for illustrative purposes. Inset indicates the positions of L5 and L3 somata, distributed uniformly and marked as dots in (**a**) the extruded slab model and (**b**) anatomically realistic head model.

Two kinds of neuronal models were distributed uniformly within the region of interest (ROI) of 50 × 50 × 50 mm^3^ with the electrodes located in the middle of each of the two models. In the extruded slab model, because of its symmetric geometry, the neuronal models were placed from the crown in the precentral gyrus to the opposite crown in the post-central gyrus along the path of the central sulcus, spaced 1 mm apart (Fig. 7 inset). Therefore, in the transverse cross-section (xy-plane), 57 neuronal models for each layer (L5 or L3) and a total of 2,907 neuronal models each (L5 or L3) were constructed. As shown in Fig. 7(a), the axons of L5 neurons were bent partially beyond the boundary between the GM and WM, and each soma of the L5 and L3 neuronal models was placed 0.6 mm and 1.8 mm above the boundary between the GM and WM^16^,^63^. In the anatomically realistic head model, two types of L5 and L3 neuronal models were constructed with configurations equivalent to those in the extruded slab model. Due to the model’s complex geometry, each neuronal model was allocated to each triangular face comprising the surface of the GM within the ROI. Therefore, a total 12,824 models for L5 and L3 neurons were modeled; the details are described in Seo *et al*.^26^.

## Additional Information

**How to cite this article**: Seo, H. *et al*. Effect of Anatomically Realistic Full-Head Model on Activation of Cortical Neurons in Subdural Cortical Stimulation—A Computational Study. *Sci. Rep.* **6**, 27353; doi: 10.1038/srep27353 (2016).

## Figures and Tables

**Table 1:** The minimum threshold (mA) for each polarity in comparison between the extruded slab model and the anatomically realistic head model.

Polarity	Extruded slab model		Anatomically realistic head model	
	L5 neurons	L3 neurons	L5 neurons	L3 neurons
anodal	7 (C)	3 (C)	8 (C)	3 (C)
cathodal	21 (C)	12 (C)	13 (B)	11 (B)
bipolar	7 (C)	3 (C)	8 (C)	3 (C)

Parentheses indicate the location of the neuron(s) excited.
